# Novel Nasal Epithelial Cell Markers of Parkinson’s Disease Identified Using Cells Treated with α-Synuclein Preformed Fibrils

**DOI:** 10.3390/jcm9072128

**Published:** 2020-07-06

**Authors:** Hyojung Kim, Seok-Jae Kang, Young Mi Jo, Sanggyu Park, Seung Pil Yun, Yun-Song Lee, Hee-Tae Kim, Nae-Eung Lee, Yong-Sang Kim, Seok Hyun Cho, Yunjong Lee

**Affiliations:** 1 Department of Pharmacology, Samsung Biomedical Research Institute, Sungkyunkwan University School of Medicine, Suwon 16419, Korea; hjung93@skku.edu (H.K.); yslee@skku.edu (Y.-S.L.); 2 Department of Neurology, H+ Yang-Ji Hospital, Seoul 08779, Korea; go2dream@hanmail.net; 3 Department of Otorhinolaryngology-Head and Neck Surgery, Hanyang University College of Medicine, Seoul 04763, Korea; jym1358@hanmail.net (Y.M.J.); hoho2027@naver.com (S.P.); 4 Department of Pharmacology and Convergence Medical Science, College of Medicine, Gyeongsang National University, Jinju 52727, Korea; spyun@gnu.ac.kr; 5 Department of Neurology, Hanyang University College of Medicine, Seoul 04763, Korea; kimht@hanyang.ac.kr; 6 Department of Advanced Materials Science and Engineering, Sungkyunkwan University, Suwon 16419, Kyunggi-do, Korea; nelee@skku.edu; 7 Department of Electrical and Computer Engineering, Sungkyunkwan University, Suwon 16419, Korea; yongsang@skku.edu

**Keywords:** nasal epithelial cell model, Parkinson’s disease, RPMI-2650 cells, olfactory biomarker, α-synuclein preformed fibril, olfactory dysfunction

## Abstract

Parkinson’s disease (PD) is the most common neurodegenerative movement disorder, characterized by olfactory dysfunction in the early stages. α-Synuclein pathologies in the olfactory organs are shown to spread to the brain through the nose-brain axis. We first developed a nasal epithelial PD cellular model by treating RPMI-2650 cells with α-synuclein preformed fibrils (PFF). Upon uptake of PFF, RPMI-2650 cells showed mitochondrial proteome alteration and downregulation of parkin, which has previously been identified as a nasal biomarker of PD. Functional cluster analysis of differentially expressed genes in RPMI-2650 cells revealed various pathways affected by α-synuclein pathology, including the detection of chemical stimulus involved in sensory perception, olfactory receptor activity, and sensory perception of smell. Among genes that were most affected, we validated, by real-time quantitative PCR, the downregulation of *MAP3K8*, *OR10A4*, *GRM2*, *OR51B6*, and *OR9A2,* as well as upregulation of *IFIT1B*, *EPN1, OR1D5, LCN, and OTOL1* in PFF-treated RPMI-2650 cells. Subsequent analyses of clinical samples showed a downregulation of *OR10A4* and *OR9A2* transcripts and an upregulation of *IFIT1B* in cells isolated from the nasal fluid of PD patients, as compared to those from the controls (cutoff value = 0.5689 for *OR9A2,* with 72.4% sensitivity and 75% specificity, and 1.4658 for *IFIT1B,* with 81.8% sensitivity and 77.8% specificity). Expression levels of these nasal PD markers were not altered in nasal fluid cells from SWEDD (scans without evidence of dopaminergic deficits) patients with PD-like motor symptoms. These nasal markers were significantly altered in patients of PD with hyposmia compared to the control hyposmic subjects. Our results validated the α-synuclein-treated nasal epithelial cell model to identify novel biomarkers for PD and suggest the utility of olfactory transcripts, along with olfactory dysfunction, in the diagnosis of PD.

## 1. Introduction

Parkinson’s disease (PD) is pathologically characterized by Lewy body inclusions, mainly composed of phosphorylated and misfolded α-synuclein aggregates [1,2]. Lewy body pathologies propagate to different brain regions in PD patients, correlating with the progressive manifestation of nonmotor and motor clinical symptoms [3]. Among several nonmotor symptoms, olfactory dysfunction develops at relatively early stages of PD, many years before the canonical motor symptoms appear. Notably, postmortem olfactory bulb tissues from several patients of PD have shown atrophic changes and the presence of α-synuclein pathologies [4,5,6]. Moreover, an injection of preformed fibrils (PFF) of α-synuclein into the olfactory bulb led to olfactory dysfunction and the subsequent propagation of Lewy-like pathology into different regions of the brain in mice [7]. Although olfactory dysfunction is known to develop in the majority of PD patients and, therefore, may serve as a useful measure for early PD diagnosis, its limitation is that olfactory dysfunctions could be induced by various factors, in many different neurodegenerative diseases and infectious diseases [5,8,9,10]. To convert a diagnosis of clinical olfactory dysfunction into the specific diagnosis of PD, additional molecular biomarkers specific to PD are required.

We have recently reported the transcripts of nasal biomarkers that are markedly altered in cells collected from the nasal fluid of patients with PD [11]. Hypothesis-driven small-scale screening identified PD-associated genes *PARKIN* and *AIMP2* as potential biomarkers for the diagnosis of PD [11]. Although it is not clear how *PARKIN* or *AIMP2* is dysregulated in these cells, easy accessibility to nasal fluid and high sensitivity afforded by PCR amplification made these nasal transcripts useful biomarkers for the diagnosis of PD. However, unbiased screening using relevant nasal cell models would provide additional biomarkers for PD, as well as mechanistic insights into the dysregulation of these transcripts. PD-related cellular and animal models have been generated with PD-linked genetic mutations or by applying mitochondrial toxins (1-methyl-4-phenyl-1,2,3,6-tetrahydropyridine (MPTP) or rotenone) or oxidant stresses (6-hydroxydopamine, H_2_O_2_, etc.) to recapitulate the major pathologies of PD [12,13]. Recently, α-synuclein preformed fibrils (PFF) have been widely used to mimic Lewy body-like inclusion in cells and in vivo [7,14,15]. PFF treatment to the cell and its uptake is sufficient to trigger misfolding and the aggregation of endogenous α-synuclein [15]. Moreover, α-synuclein pathologies in certain brain regions can be propagated to other parts through α-synuclein transmission. Since α-synuclein pathologies can be transmitted, and Lewy pathologies have been reported in olfactory organs [4], it is plausible that olfactory systems could be influenced by α-synuclein pathologies in PD. Therefore, PFF could be applied to develop a cellular model of PD, using nasal epithelial cells, to screen potential PD-related nasal markers.

Here, we developed a novel nasal epithelial cell model of PD by treating RPMI-2650 cells with PFF. Using this model, we screened several α-synuclein pathology-regulated markers that were subsequently validated in cells obtained from the nasal fluid of PD patients. We found that the expressions of olfactory receptor 10A4 (*OR10A4*), *OR9A2*, and interferon-induced protein with tetratricopeptide repeats 1B (*IFIT1B*) were significantly altered in PD compared to the control. Even among hyposmia patients, these markers were extensively altered only in PD, suggesting their potential in diagnosing PD in patients with olfactory dysfunction.

## 2. Materials and Methods

### 2.1. Subjects

To evaluate the nasal expression of PD-related markers, 23 PD patients, 8 scans without evidence of dopaminergic deficits (SWEDD) patients and 13 age-matched controls were included (Table 1). All patients with PD were diagnosed according to the United Kingdom Brain Bank Diagnostic Criteria [16,17]. SWEDD patients were diagnosed by PD-like clinical motor symptoms with a normal 18F-N-(3-fluoropropyl)-2β-carboxymethoxy-3β-(4-iodophenyl) nortropane (18F-FP-CIT) positron emission tomography (PET) scan for the dopamine transporter in the striatum. The diagnosis of olfactory functions was done according to established criteria, and the cases were classified as anosmia (0–15), hyposmia (16–29), and normosmia (≥30) according to threshold-discrimination-identification (TDI) scores [18]. All patients were investigated by specialists of movement disorders and olfaction to keep the risk of misdiagnosis at a minimum. Nasal fluid samples were collected from PD SWEDD patients and the controls (with no symptoms or history of neurodegenerative diseases) when they visited the Department of Otolaryngology. No significant differences in the distribution of cases based on sex (*p* = 0.6251) and age (*p* = 0.3356) were seen among the three groups. Written informed consent was obtained from all participants prior to the start of the investigation. This study was approved by the Institutional Review Board of Hanyang University Medical Center (No. 2018-01-002-002), and all experiments involving human subjects and samples were performed in accordance with relevant guidelines and regulations.

### 2.2. Nasal Fluid Sampling

Nasal fluids were collected through a suction collector following two puffs of sterile normal saline solution into each nasal cavity (total 10 μL) and transferred immediately to the laboratory bench. After the removal of crust and debris with a cell strainer (100 μm, SPL, Pocheon, Gyeonggi-do, South Korea), the fluids were centrifuged at 6000 rpm for 5 min. The cell pellets were resuspended in 1 mL of TRIzol and stored at −80 °C until analysis.

### 2.3. Total RNA Extraction and PCR

Total RNA was extracted from the cells collected from the nasal fluid using the QIAzol Lysis Reagent (cat# 79306, Qiagen, Hilden, Germany) by adding carrier yeast tRNA (10 µg) followed by DNase I treatment to eliminate any trace of DNA contamination. Complementary DNA (cDNA) was synthesized from the purified nasal cell RNA with the First-Strand cDNA Synthesis kit (#170-8891, iScript cDNA synthesis kit, Bio-Rad, Hercules, CA, USA). Cycle threshold (Ct) values of each gene were obtained from the SYBR green-based real-time PCR reaction using the QuantStudio 6 Flex Real-Time PCR System (Applied Biosystems, Foster city, CA, USA). Relative messenger ribonucleic acid (mRNA) levels of target genes were calculated through the delta-delta-cycle threshold (ΔΔCt) method [19] using GAPDH or β-actin as the internal loading control. The SYBR Green PCR Master Mix (Cat# 4309155, Applied Biosystems) was used according to the manufacturer’s instructions. The primers utilized for real-time PCR amplification are summarized in Table 2.

### 2.4. Cell Culture and PFF Treatment

Human nasal epithelial RPMI-2650 cells (ATCC#CCL-30, ATCC, Manassas, VA, USA) were grown in eagle’s minimum essential medium (EMEM) (ATCC#30-2003) containing 10% fetal bovine serum (FBS) (v/v) and antibiotics (penicillin–streptomycin 100 U/mL; Sigma-Aldrich, St. Louis, MO, USA). Cells were propagated in a humidified atmosphere consisting of 5% CO_2_/95% air and maintained at 37 °C. These cells were plated in 6-well plates at a density of 0.5 × 10^6^ cells per well and harvested at 72 h after treatment with PFF (final concentration (F.C.); 10 µg/mL) or phosphate-buffered saline (PBS).

### 2.5. Western Blotting

SH-SY5Y cells were briefly washed with ice-cold PBS, and total protein lysates were prepared in the lysis buffer (1% Nonidet P40 in phosphate-buffered saline (PBS), pH 7.4) supplemented with protease/phosphatase inhibitors. After three freeze and thaw cycles in dry ice, total protein lysates were centrifuged at 14,000× *g* for 30 min. The supernatants were mixed with 2× Laemmli buffer (Bio-Rad) supplemented with β-mercaptoethanol (Sigma-Aldrich, St. Louis, MO, USA) and boiled for 5 min. Proteins were separated by sodium dodecyl sulfate polyacrylamide gel electrophoresis (SDS-PAGE) and transferred onto nitrocellulose membranes for immunoblotting. Immunoblotting was performed by incubation of the membrane in the indicated primary antibodies (mouse antibody to parkin (cat#4211, 1:5000; Cell Signaling Technology, Danvers, MA, USA), rabbit antibody to pyruvate dehydrogenase (cat#3205, 1:5000; Cell Signaling Technology), rabbit antibody to the voltage-dependent anion channel (VDAC) (cat#4661, 1:5000; Cell Signaling Technology), rabbit antibody to prohibitin 1 (PHB1) (cat#2426, 1:5000; Cell Signaling Technology), and rabbit antibody to cytochrome c oxidase subunit 4 (COX IV) (cat#4850, 1:5000; Cell Signaling Technology), followed by horseradish peroxidase (HRP)-conjugated secondary antibodies (peroxidase (HRP)-conjugated goat antibody to mouse immunoglobulin G (IgG) (cat# GTX-213111-01, 1:5000; Genetex, Irvine, CA, USA) and HRP-conjugated goat antibody to rabbit IgG (cat# GTX-213110-01, 1:5000; Genetex). The bands were visualized via chemiluminescence (Pierce). Densitometric analysis of the bands was performed using ImageJ software 1.52v (NIH; http://rsb.info.nih.gov/ij/).

### 2.6. Raw Data Preparation

The data were summarized and normalized using the robust multi-average (RMA) method implemented in Affymetrix^®^ Power Tools (APT 2.11.2). We exported the results with gene-level RMA analysis and analyzed differentially expressed genes (DEGs). Those genes with reproducible results and showing more than 30% alteration in the expression level in two independent experiments were used for subsequent DEG analysis. For a DEG set, a hierarchical cluster analysis was performed using complete linkage and Euclidean distance as a measure of similarity. Gene enrichment and functional annotation analysis for the selected list of genes were performed using Cytoscape GlueGO (Gene Ontology, biological process). In total, 688 genes were recognized by ClueGO, and 230 genes were functionally annotated by biological process ontology. Genes from all clusters were associated with 8 representative terms and pathways with a *p*-value of 0.01 corrected with the Bonferroni step down method. The minimum percentage of associated genes to total genes in each functional cluster was set to 4%. The details of the Cytoscape ClueGO analysis can be found in Supplementary Table S1.

### 2.7. Statistical Analyses

Quantitative data are presented as the mean ± standard error of the mean (SEM). Normality of the data was tested with the Shapiro–Wilk test. Statistical significance was assessed via the nonparametric Mann–Whitney U test for a two-group comparison. Correlation was determined with the Pearson correlation analysis. All assessments were considered statistically significant when the *p*-value was lower than 0.05.

## 3. Results

### 3.1. Generation of PFF-Induced Nasal Cell Model of PD

**Figure 1:**
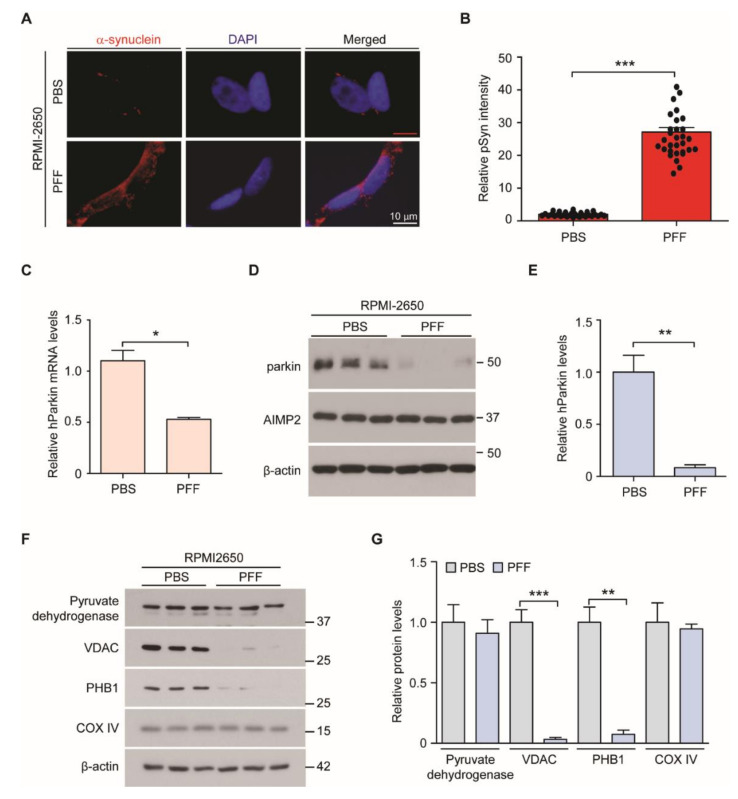
The generation of a novel preformed fibril (PFF)-induced nasal epithelial cellular model of Parkinson’s disease (PD). (**A**) Representative immunofluorescence images showing PFF uptake (10 µg/mL PFF treatment for 3 days) by RPMI-2650 nasal epithelial cells using anti-α-synuclein-specific antibodies. (**B**) Quantification of relative α-synuclein immunofluorescence intensities in RPMI-2650 cells with the PFF treatment (10 µg/mL, 3 days) or without the PFF treatment (*n* = 30 cells per group from three independent experiments). (**C**) Quantification of relative *PARKIN* messenger ribonucleic acids (mRNA) levels by reverse-transcription quantitative PCR in RPMI-2650 cells treated with phosphate-buffered saline (PBS) or PFF (10 µg/mL, 3 days) normalized to *GAPDH* as the internal loading control (*n* = 3 per group). (**D**) Representative Western blots of PARKIN in RPMI-2650 cells treated either with PBS or PFF, using the indicated antibodies. (**E**) Quantification of the *PARKIN* expression in the indicated experimental groups normalized to β-actin (*n* = 3 per group). (**F**) Representative Western blots of pyruvate dehydrogenase, voltage-dependent anion channel (VDAC), prohibitin 1 (PHB1), and cytochrome c oxidase subunit 4 (COX IV) in RPMI-2650 cells treated either with PBS or PFF, using the indicated antibodies. (**G**) Quantification of pyruvate dehydrogenase, VDAC, PHB1, and COX IV expressions in the indicated experimental groups normalized to β-actin (*n* = 3 per group). The data are expressed as the mean ± SEM. * *p* < 0.05, **p < 0.01, and *** *p* < 0.001, nonparametric Mann–Whitney U test.

The effects of the PFF treatment mimic intracellular α-synuclein aggregation and PD-related pathologies in neuron cultures and in vivo [7,14,15]. Similarly, to develop a model of PD using nasal epithelial cells, we treated the nasal epithelial cells, RPMI-2650, with PFF. This led to the robust accumulation of intracellular α-synuclein-positive signals (Figure 1A,B), indicating the efficient uptake of PFF. To characterize this novel model of PD, we examined the transcript levels of *PARKIN*, a previously validated nasal biomarker of PD [11]. For this, the total RNA was extracted from cells that were treated with or without PFF. Real-time quantitative PCR (RT qPCR) analysis revealed an approximate 50% reduction in *PARKIN* transcripts in the PFF-treated RPMI-2650 cell line (Figure 1C). Western blots using anti-PARKIN antibody showed a marked reduction of PARKIN expression in these PFF-induced cells (Figure 1D,E). Moreover, Western blot analysis of mitochondrial proteins revealed a marked downregulation of the voltage-dependent anion channel (VDAC) and prohibitin 1 (PHB1) (Figure 1F,G). However, there was no alteration in the expression of pyruvate dehydrogenase and cytochrome c oxidase subunit 4 (COX IV) (Figure 1F,G). These results indicate that the PFF treatment affects gene expression profiles and mitochondrial proteome in RPMI-2650 cells, thereby suggesting its utility for screening potential nasal PD biomarkers derived from nasal epithelial cells.

### 3.2. Screening of PD Biomarkers in the PFF-Exposed Nasal Cell Model

**Figure 2:**
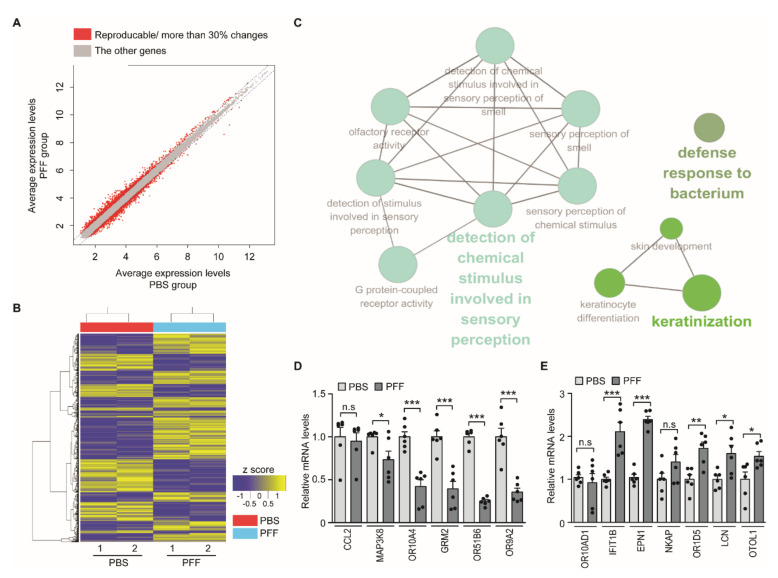
The PFF treatment of nasal epithelial cells alters the expression of olfaction-related genes. (**A**) Expression level plots of genes in the PBS (x-axis) and PFF (y-axis) groups determined by microarray analysis. Those genes with reproducible and more than 30% alterations in two independent experiments are shown in red, and only these genes were used for subsequent analyses. (**B**) Hierarchical cluster analysis of genes differentially expressed in PFF-treated RPMI-2650 cells (*n* = 2 per group) determined by complete linkage and Euclidean distance as a measure of similarity. (**C**) Functional pathway clustering of the selected genes with reproducible and more than 30% alterations between PBS- and PFF-treated RPMI-2650 cells analyzed by the Cytoscape software ClueGO plugin. (**D**,**E**) Quantification and validation of selected gene transcripts from PFF-treated RPMI-2650 cells with alterations in the microarray analysis determined by reverse-transcription quantitative PCR (upregulated genes in the microarray: *CCL2*, *MAP3K8*, *OR10A4*, *GRM2*, *OR51B6*, and *OR9A2*; downregulated genes in the microarray: *OR10AD1*, *IFIT1B*, *EPN1, NKAP, OR1D5, LCN,* and *OTOL1*. *n* = 6 per group). The data are expressed as the mean ± SEM * *p* < 0.05 **p < 0.01, and *** *p* < 0.001, nonparametric Mann–Whitney U test. n.s, nonsignificant.

To identify PFF-induced alterations in the transcriptome of RPMI-2650 cells, we extracted total RNA from PFF-treated cells or PBS-treated controls. From two independent microarray experiments, we further analyzed the genes that showed reproducible and greater than 30% changes in their expression levels after PFF treatment (Figure 2A and Supplementary Table S2). A heatmap analysis with hierarchical clustering revealed these groups of genes whose expressions changed in response to the PFF treatment (Figure 2B). Functional clustering by Cytoscape ClueGo showed the enrichment of these genes in several biological functional categories, including the detection of chemical stimulus involved in the sensory perception (of smell), olfactory receptor activities, and the sensory perception of smell and chemical stimulus (Figure 2C). Other functional categories were a defense response to keratinization (Figure 2C). This bioinformatic analysis of differentially expressed genes (DEGs) suggests the potential dysregulation of olfactory functions in RPMI-2650 epithelial cells by PFF.

Next, we sought to validate the top-ranked genes in the microarray (Table 3) with RT qPCR using specific primer sets (Table 2). We used GAPDH as an internal loading control, since there was no observable alteration of GAPDH expression in response to PFF treatment when normalized to β-actin ( Supplementary Figure S1A). Barring the motif chemokine ligand 2 (*CCL2*), the other five genes—mitogen-activated protein kinase kinase kinase 8 (*MAP3K8*), olfactory receptor 10A4 (*OR10A4*), glutamate metabotropic receptor 2 (*GRM2*), olfactory receptor 51B6 (*OR51B6*), and olfactory receptor 9A2 (*OR9A2*)—were downregulated by more than 50% (Figure 2D and Table 1) in PFF-treated RPMI-2650 cells compared to the PBS-treated control cells (Figure 2D). Genes that were upregulated in the microarray analysis (Table 1)—olfactory receptor 10AD1 (*OR10AD1*), *IFIT1B*, epsin 1 (*EPN1*), NFKB activating protein (*NKAP*), olfactory receptor 1D5 (*OR1D5*), lipocalin (*LCN*), and otolin 1(*OTOL1*)—were also validated by RT qPCR. We observed a marked increase in the transcript levels of *IFIT1B* and *EPN1* in PFF-treated RPMI-2650 cells compared to the control (Figure 2E). There was a modest increase in the transcript levels of OR1D5, LCN, and OTOL1 (Figure 2E).

### 3.3. Quantitative Assessment of PD Marker Gene Transcripts in Cells Obtained from Clinical Nasal Fluid Samples

For the translational application of novel disease markers identified from the novel nasal cell model of PD, nasal fluid samples were collected from 23 PD patients: eight scans without evidence of dopaminergic deficits (SWEDD) patients and 13 age-matched healthy controls (Table 1). There was no significant difference (*p* = 0.3356) in the age distribution among the PD (average 67 years), SWEDD (average 80 years), and control (average 63 years) groups. We included both male and female subjects within the PD, SWEDD, and the control group in the study (*p* = 0.6251). The evaluation and diagnosis of patients were performed by an experienced neurologist based upon the Hoehn and Yahr (H&Y) scale (range, 1–5) and the unified PD rating score (UPDRS; range, 5–62). The average values based on the H&Y scale and UPDRS score in the PD and control groups were 2.30 and 26.4, respectively (Table 3). Olfactory functions, evaluated by an experienced otolaryngologist by using threshold-discrimination-identification (TDI) scoring, were classified as normal (TDI score: ≥30), hyposmia (TDI score: 16–29), and anosmia (TDI score: 0–15) cases. The numbers of normal olfaction, hyposmia, and anosmia cases were 5, 13, and 5, respectively, in the PD group; one, five, and two for the SWEDD group; and six, seven, and zero, respectively, in the control group.

**Figure 3:**
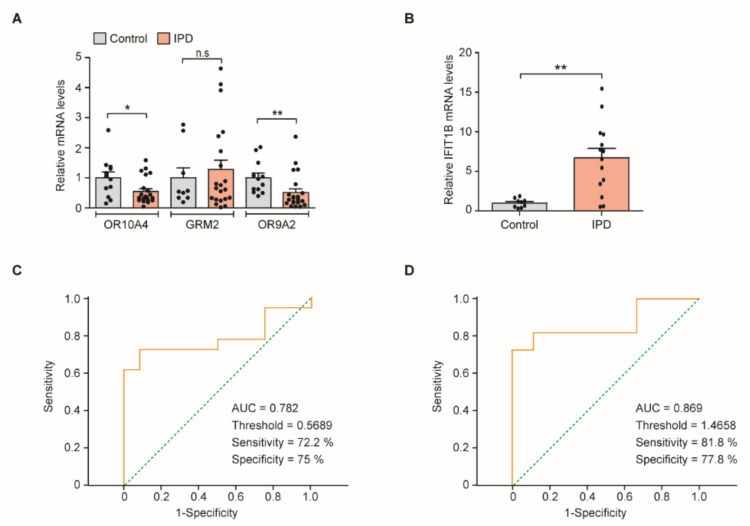
Quantitative analysis of *OR10A4*, *OR9A2*, *GRM2*, and *IFIT1B* transcripts in the cells obtained from human nasal fluid cells. (**A**,**B**) *OR10A4*, *OR9A2*, *GRM2*, and *IFIT1B* in the cells obtained from the nasal fluid of PD patients and healthy controls determined by reverse-transcription quantitative PCR followed by normalization to the internal loading control *GAPDH* (*n* = 12 control (con) and 22 PD for *OR10A4* and *GRM2* analysis, 10 con and 22 PD for *OR9A2* analysis, and 9 con and 14 PD for *IFIT1B* analysis). (**C**,**D**) Receiver operating characteristic curve analysis of sensitivity and specificity for *OR9A2* and *IFIT1B* as biomarkers of PD. The data are expressed as the mean ± SEM. * *p* < 0.05 and ** *p* < 0.01; nonparametric Mann–Whitney U test. AUC: area under the curve. n.s, nonsignificant.

Nasal fluid cells were collected for total RNA extraction from these enrolled PD patients and age-matched controls. We monitored relative levels of the transcripts of potential markers of PD (*MAP3K8*, *OR10A4*, *GRM2*, *OR51B6*, *OR9A2*, *IFIT1B*, and *EPN1*) that were validated and showed marked changes in expressions in the in vitro experiments. Target genes were amplified using specific primer sets (Table 2), and cDNA templates were prepared using total RNA from the nasal cell samples of the patients and controls. The relative amounts of these target genes were determined by the ΔΔCt method [19] and normalized to the *GAPDH* expression as an internal loading control. Similar to RPMI-2650 cells treated with PFF, nasal fluid cells from both PD and the age-matched control showed comparable GAPDH expressions when normalized to β-actin (Supplementary Figure S1B), justifying the use of GAPDH as an internal loading control. Among the seven target genes, only four genes (*OR10A4*, *GRM2*, *OR9A2*, and *IFIT1B*) were successfully amplified, whereas *MAP3K8*, *OR51B6*, and *EPN1* could not be amplified, probably due to their low expression levels. Real-time assessment showed a significant downregulation of *OR10A4* and *OR9A2* (45% and 50%, respectively) in the PD group compared to the control (Figure 3A). This result was consistent with our in vitro findings. However, *GRM2* expression did not significantly differ between the PD and control groups (Figure 3A). *IFIT1B* was upregulated (6.7-fold) in the PD group compared to the control, similar to our in vitro findings (Figure 3B). *IFIT1B* expression within the PD group significantly varied, while its levels were maintained consistently low in the control group (Figure 3B). To determine whether OR10A4, OR9A2, and IFIT1B alterations are PD-specific, we monitored the expression levels of these transcripts in the SWEDD patients. The results showed that the nasal transcripts levels were not changed in the SWEDD group as compared to the age-matched control (Supplementary Figure S2).

We also evaluated the expression of four target genes (OR10A4, OR9A2, GRM2, and IFIT1B) during the progression of PD. The expression of *OR10A4*, *OR9A2*, *GRM2*, and *IFIT1B* did not show any dependency on the H&Y scale (score ranges: 0, 1–2.5, or 3–5) (Supplementary Figure S3A–D) or UPDRS scores (Supplementary Figure S4A–D), indicating that these markers are poor indicators of PD severity. However, the transcript levels of *OR10A4* and *OR9A2* strongly and inversely correlated with the *IFIT1B* transcript levels (Supplementary Figure S5A,B), suggesting that these markers can be used in combination to improve PD diagnosis.

### 3.4. Receiver Operating Characteristic (ROC) Curve Threshold Analysis of OR9A2 and IFIT1B mRNA Levels for PD Diagnosis

To evaluate the clinical diagnostic values of *OR10A4*, *OR9A2*, and *IFIT1B*, whose expression was significantly altered in PD, we applied a ROC curve analysis. The ROC curve analysis of *OR10A4* failed to produce a significant outcome with an area under the curve (AUC) value of 0.681 and a *p*-value of 0.099. However, the AUC for *OR9A2* was 0.782 (Figure 3C), with a *p*-value of 0.01, indicating that the *OR9A2* level can be used for PD diagnosis. With a cutoff threshold of 0.5689, the sensitivity and specificity of PD diagnosis by *OR9A2* were 72.2% and 75%, respectively (Figure 3C). The ROC curve analysis of *IFIT1B* revealed an AUC value of 0.869 and a *p*-value 0.006, showing that it can be used in PD diagnosis. With a cutoff threshold value of 1.4658, the sensitivity and specificity of PD diagnosis by *IFIT1B* were 81.8% and 77.8%, respectively (Figure 3D).

### 3.5. Quantitative Assessment of Transcripts of PD Marker Genes in Hyposmia Cases

**Figure 4:**
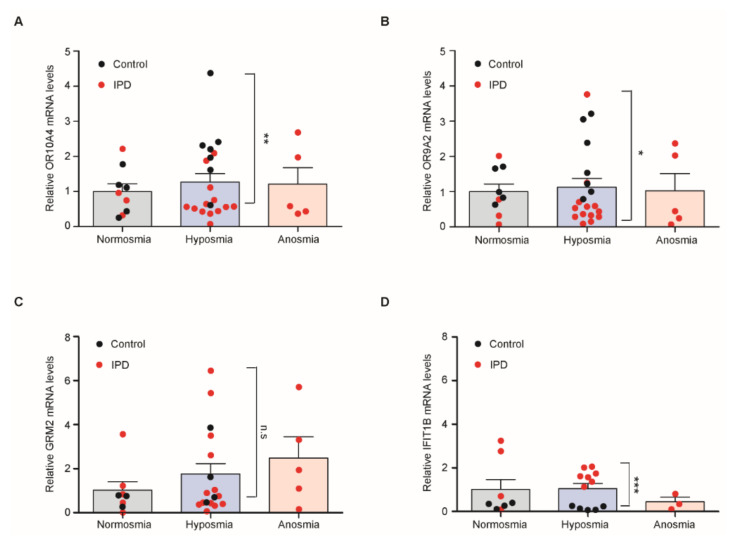
Analysis of nasal transcripts between normosmia, anosmia, and hyposmia cases in the PD and control groups. (**A**) Relative nasal *OR10A4* transcript levels in three groups of different olfactory statuses (*n* = 9 normosmia, 20 hyposmia, and 5 anosmia). PD and controls are expressed as red and black filled circles (*n* = 12 con and 22 PD), respectively. (**B**) Relative nasal *OR9A2* transcript levels in three groups of different olfactory statuses (*n* = 9 normosmia, 20 hyposmia, and 5 anosmia). PD and controls are expressed as red and black filled circles (*n* = 12 con and 22 PD), respectively. (**C**) Relative nasal *GRM2* transcript levels in three groups of different olfactory statuses (*n* = 8 normosmia, 17 hyposmia, and 5 anosmia). PD and controls are expressed as red and black filled circles (*n* = 7 con and 23 PD), respectively. (**D**) Relative nasal *IFIT1B* transcript levels in three groups of different olfactory statuses (*n* = 8 control, 12 hyposmia, and 3 anosmia). PD and controls are expressed as red and black filled circles (*n* = 9 con and 14 PD), respectively. The data are expressed as the mean ± SEM. Statistical comparison was done for the hyposmia cases in the PD and control groups. * *p* < 0.05, ** *p* < 0.01, and *** *p* < 0.001. n.s, nonsignificant.

Since the markers identified previously in our in vitro analysis and in clinical samples revealed dysregulation of olfaction-related pathways, we analyzed the expression of these markers after regrouping the enrolled subjects by olfactory functions. PD and control subjects were categorized to normal olfactory functions (normosmia group with TDI over 30), reduced olfactory functions (hyposmia with TDI range 16–29), and severely impaired olfactory functions (anosmia with TDI range 0–15) (Figure 4A–D). Transcript levels of *OR10A4* and *OR9A2* were substantially lower, while those of *IFIT1B* were significantly higher in cases of hyposmia in the PD group than the control group (Figure 4A,B,D). In contrast, *GRM2* mRNA levels were comparable between hyposmia cases in the two groups (Figure 4C). These results indicate that *OR10A4*, *OR9A2*, and *IFIT1B* exhibited PD-specific alterations, reflecting the potential of these markers to distinguish hyposmia and hyposmia that could develop into PD.

## 4. Discussion

In this study, we first developed a novel cellular model of PD using nasal epithelial cells, which could reflect pathological genetic changes in response to α-synuclein aggregates. PFF has been widely employed to trigger α-synucleinopathy-induced cellular dysfunction in neurons to model PD [15,20]. It can influence diverse cell populations such as microglia, in addition to neurons, in the brain [21,22]. α-synuclein pathologies can be transmitted to many different brain regions via receptor-mediated or nonreceptor-mediated mechanisms [23,24]. Since α-synuclein aggregates are found in the body fluids of PD patients and olfactory dysfunction appears in most PD patients in the early stages, we considered that α-synuclein may impact the nasal environment. We previously reported that transcript levels of *PARKIN* or *AIMP2* in the nasal cells of PD patients can serve as excellent diagnostic markers [11]. Although cells from the nasal fluid are composed of epithelial cells, squamous cells, and inflammatory cells, we developed a nasal cell model of PD using RPMI-2650 cells that are derived from an anaplastic squamous cell carcinoma of the nasal septum [25,26] by exposing them to PFF. We screened potential PD-associated nasal genes whose expression levels were regulated by α-synuclein. We found that *PARKIN* was significantly downregulated at the transcript and protein levels in PFF-induced RPMI-2650 cells and in nasal cells obtained from PD patients. The PFF treatment in RPMI-2650 cells resulted in alterations in mitochondrial protein expressions, such as VDAC and PHB1. Interestingly, the downregulation of both proteins is implicated in PD pathogenesis [27,28]. Our nasal cell model of PD may be useful in PD diagnosis, because the DEGs identified in this model are involved in olfaction. It is largely unknown whether those gene clusters in olfactory biological functions are also dysregulated in olfactory neurons that are functional units of olfactory perceptions. An immunohistological or in situ hybridization study is required to determine the expression pattern of olfactory-related genes in the nasal epithelium of PD. Nevertheless, the downregulation of two olfaction-related genes, *OR10A4* and *OR9A2*, was validated in our in vitro and clinical experiments. Our results suggest the usefulness of applying PFF to model PD in diverse cell lines, including neurons, and screen potential markers of PD.

*PARKIN* is a recessive PD-linked gene encoding neuroprotective E3 ubiquitin ligase. Its biological functions include proteasomal degradation of pathologic substrates (e.g., AIMP2 and PARIS), receptor trafficking, gene expression, and mitophagy [2]. The transcriptional regulation of *PARKIN* can be influenced by endoplasmic reticulum stress [29] or glucocorticoid receptor activation [30]. Moreover, the promoter of *PARKIN* can be methylated to regulate its transcription [31]. It is not clear how PFF represses *PARKIN* transcription in RPMI-2650 cells. The dysregulation of the aforementioned mechanisms by PFF could impact *PARKIN* expression. It is also possible that α-synuclein aggregates sequester biologically important transcriptional regulators [32] and affect *PARKIN* expression. While our microarray analysis failed to detect a downregulation of *PARKIN* transcripts in PFF-treated RPMI-2650 cells, RT qPCR analysis confirmed the downregulation. This discrepancy could be due to the variations of sensitivity or specificity of relevant *PARKIN* probes in the microarray chips. However, among the top-ranked nine genes, microarray results for the alterations in the expressions of seven genes were consistent with those of RT qPCR (Figure 2D).

*OR9A2* and *IFIT1B* were found to be efficient biomarkers for PD. It was surprising to observe a marked alteration in the expression of olfactory receptors in RPMI-2650 cells, as olfactory receptors are mainly expressed in olfactory nerve terminals located on the nasal epithelium. Olfactory receptors are the largest member of the G protein-coupled receptors and are important for detecting diverse chemical stimuli in the nasal epithelium. However, the expression of olfactory receptors in nonchemosensory tissues, especially in the testis, sperm, and ventral midbrain, has been noted [33,34]. Similarly, several classes of olfactory receptors with distinct functions may be expressed in RPMI-2650 cells, which are derived from the nasal epithelium. Many olfactory receptors are expressed in dopaminergic neurons in the ventral midbrain of mice and humans [33]. However, the mechanism of PFF-induced alteration in *OR9A2* expression in RPMI-2650 cells is unknown. Moreover, *OR9A2* was downregulated in the nasal cells obtained from PD patients. As this dysregulation of the expression of olfactory receptors may be associated with olfactory dysfunction in PD, a mechanistic analysis of its regulation may shed light on the molecular mechanisms of olfactory dysfunction associated with PD. *IFIT1B* showed excellent sensitivity and specificity in PD diagnosis. *IFIT1B* expression can be stimulated by interferons during viral infections [35]. Its role in the antiviral host defense is mediated by the inhibition of viral replication through binding to and regulating the cellular and viral RNA and proteins [35]. The mechanism of a PFF-induced enhancement in the *IFIT1B* expression in both PFF-treated RPMI-2650 cells and nasal cells obtained from PD patients is unknown. However, it is interesting to note that a prion infection increases interferon responsive mRNA in Creutzfeldt-Jakob disease [36], and interferons have been shown to suppress prion infection [37]. This suggests the potential role of interferon in downstream pathways in response to α-synuclein aggregation, which possess similar properties to prion infection. It would be instructive to further investigate interferon regulatory signaling in PD diagnosis and, also, therapeutic targets in PD with α-synuclein pathology propagation

Olfactory functions are impaired in early PD. Therefore, an olfactory functions test may be valuable in PD diagnosis. Moreover, since olfactory abnormalities appear 5–10 years before canonical motor symptoms develop [5], they can be used to diagnose PD in its prodromal stages. However, olfactory dysfunctions can occur due to viral infection, allergen-induced inflammation, traumatic injuries, or neurodegenerative pathologies [11,38,39], such as Alzheimer’s disease, in addition to PD. This heterogeneity limits the clinical application of olfaction testing as a specific diagnostic marker for PD. In this regard, our present study provided a molecular basis to supplement the currently available clinical olfactory test to differentiate PD. *OR10A4*, *OR9A2*, and *IFIT1B* hold potential in differentiating PD-related hyposmia from non-PD-related hyposmia (Figure 4A,B,D). Indeed, these three markers in nasal fluid cells were not altered in SWEDD patients, indicating a PD specificity of these nasal transcripts. SWEDD patients exhibit clinical movement symptoms that are indistinguishable from PD; however, SWEDD patients are reported to have distinct pathologic etiology from PD [40]. Since SWEDD patients do not develop progressive dopaminergic neurodegeneration, and they are not responsive to L-DOPA supplementation [40], an accurate diagnosis using imaging or biomarkers is required to differentiate SWEDD from dopamine-deficient PD. In this regard, our nasal PD biomarkers could be applicable for a better diagnosis of PD and SWEDD. It is still necessary to validate the clinical utility of these nasal PD biomarkers in a larger cohort of PD and control populations, ideally including more cases of other neurodegenerative disorders to determine if these markers are specific to PD.

## Figures and Tables

**Table 1:** Basic demographic information, clinical motor assessment, and olfactory scores of Parkinson’s disease (PD), scans without evidence of dopaminergic deficits (SWEDD) patients, and healthy controls.

	Control	PD	SWEDD	*p*-Value
*N*	13	23	8	
Male: female	6:7	9:14	2:6	0.6251
Age (years)	63 ± 2.62	67 ± 2.66	80 ± 1.88	0.3356
H&Y scale		2.30 ± 0.25		
UPDRS		26.4 ± 3.75		
Olfaction (TDI score)	29.5 ± 2.41	23.4 ± 2.07	21.3 ± 2.48	0.0887

The quantitative values are presented as the mean ± SEM. The ANOVA test was performed to compare the mean ages among the groups. Age distribution failed to show a significant difference among the groups. H&Y: Hoehn and Yahr scale, UPDRS: unified PD rating score, and TDI: threshold-discrimination-identification.

**Table 2:** Primer sequences used for the amplification of the target genes in RT qPCR.

Target Gene	Sequence (5′→3′)
*GAPDH*	Forward: AAACCCATCACCATCTTCCAG
	Reverse: AGGGGCCATCCACAGTCTTCT
*β-ACTIN*	Forward: AGAGCTACGAGCTGCCTGAC
	Reverse: AGCACTGTGTTGGCGTACAG
*MAP3K8*	Forward: CTCCCCAAAATGGACGTTACC
	Reverse: GGATTTCCACATCAGATGGCTTA
*OR10A4*	Forward: AGCTGCCTCTTGGTTCTCAG
	Reverse: ACAGACCAGTGCAATAACAGG
*GRM2*	Forward: CCGCATTGCACGCATCTTC
	Reverse: GGCCCGAGATAAGTGCCAG
*OR51B6*	Forward: ATGGGGCTCAATAAGTCTGCT
	Reverse: GAGGTCTGTAGCTGCCAACA
*OR9A2*	Forward: ATGTATCGCTCAACTTTTCCTGT
	Reverse: CCCATGACACTATTACCACCCAA
*CCL2*	Forward: CAGCCAGATGCAATCAATGCC
	Reverse: TGGAATCCTGAACCCACTTCT
*OR10AD1*	Forward: CTAAGGAATGGCAGCATAGTGAC
	Reverse: AGCAGACATCCAGGAGAGACA
*IFIT1B*	Forward: AATCAAGGAAGCTACAAACTGGC
	Reverse: TCTTCATGCGTAACCCTTTCTG
*EPN1*	Forward: CAAGAACTGGCGTCACGTTTA
	Reverse: CTGCTTAGCTTTCTCACGCA
*NKAP*	Forward: CAGTCTATTACGGCAGCTACTCG
	Reverse: CTCCACTGGTGTATGTTCATCAG
*OR1D5*	Forward: TTCACACAGCGTTGATTGCC
	Reverse: CAAGCGTCCCATAAAAGAGGG
*LCN2*	Forward: GAAGTGTGACTACTGGATCAGGA
	Reverse: ACCACTCGGACGAGGTAACT
*OTOL1*	Forward: GTCCTGTCACTGGGAAGTTTAAC
	Reverse: CCAGACTGATTCGAGCAGGTC

**Table 3:** Differentially expressed genes between the preformed fibril (PFF)-treated and phosphate-buffered saline (PBS_-treated RPMI-2650 cells (top upregulated and downregulated genes; see Table S2 for the complete list).

Gene Symbol	Gene Description	FC (Microarray)	FC (RT qPCR)
BAGE2	B melanoma antigen family, member 2	0.58	ND
TMC3	Transmembrane channel like 3	0.59	ND
MAP3K8	Mitogen-activated protein kinase kinase kinase 8	0.60	0.73
OR10A4	Olfactory receptor, family 10, subfamily A, member 4	0.61	0.42
GRM2	Glutamate receptor, metabotropic 2	0.62	0.39
KRTAP10-2	Keratin associated protein 10-2	0.63	ND
OR51B6	Olfactory receptor, family 51, subfamily B, member 6	0.63	0.25
OR9A2	Olfactory receptor, family 9, subfamily A, member 2	0.64	0.35
KLK15	Kallikrein related peptidase 15	0.64	ND
CCL2	Chemokine (C-C motif) ligand 2	0.64	0.95
GSTA2	Glutathione S-transferase alpha 2	1.91	ND
OR10AD1	Olfactory receptor, family 10, subfamily AD, member 1	1.71	0.88
OR5M1	Olfactory receptor, family 5, subfamily M, member 1	1.61	ND
DAPL1	Death associated protein like 1	1.60	ND
IFIT1B	Interferon-induced protein with tetratricopeptide repeats 1B	1.60	2.11
EPN1	Epsin 1	1.59	2.28
OR2T4	Olfactory receptor, family 2, subfamily T, member 4	1.59	ND
TSTD3	Thiosulfate sulfurtransferase (rhodanese)-like domain containing 3	1.58	ND
TAS2R43	Taste receptor, type 2, member 43	1.57	ND
SPANXN5	SPANX family, member N5	1.57	ND
NKAP	NFKB activating protein	1.44	1.40
OR1D5	olfactory receptor, family 1, subfamily D, member 5	1.46	1.72
LCN2	lipocalin 2	1.50	1.60
OTOL1	otolin 1	1.56	1.54

Based upon the microarray results, top-ranked differentially expressed genes (DEGs) were further analyzed by real-time quantitative PCR (RT qPCR). Those genes with available primers (obtained from the “Primer Bank” website) were presented with average fold changes of expression by RT qPCR in comparison to the microarray fold change values. “ND” are those genes with no available primer information in the “Primer Bank” website or no PCR amplification. FC, fold change.

## Supplementary Materials

The following are available online at https://www.mdpi.com/2077-0383/9/7/2128/s1: Figure S1. Relative GAPDH mRNA expression under PD-relevant conditions. Figure 2. Levels of potential nasal PD biomarkers in SWEDD. Figure S3. Levels of potential nasal biomarkers in different stages of PD. Figure S4. Correlation analyses of the levels of nasal OR10A4, OR9A2, GRM2, and IFIT1B and clinical UPDRS scores. Figure S5. Correlation analysis between potential nasal biomarkers. Table S1: Gene enrichment and functional annotation analysis using Cytoscape GlueGO (Gene Ontology, biological process). Table S2: The complete list of genes with reproducible and more than 30% alterations between PFF-treated and PBS-treated RPMI-2650 cells.
