# Supplementary material for: Novel Nasal Epithelial Cell Markers of Parkinson’s Disease Identified Using Cells Treated with α-Synuclein Preformed Fibrils
Source: J Clin Med. 2020 Jul 6;9(7):2128. doi: 10.3390/jcm9072128 (PMC7408990; doi:10.3390/jcm9072128)
Supplement: Supplementary file 1 [file jcm-09-02128-s001.zip › supplementary materials-final.pdf]

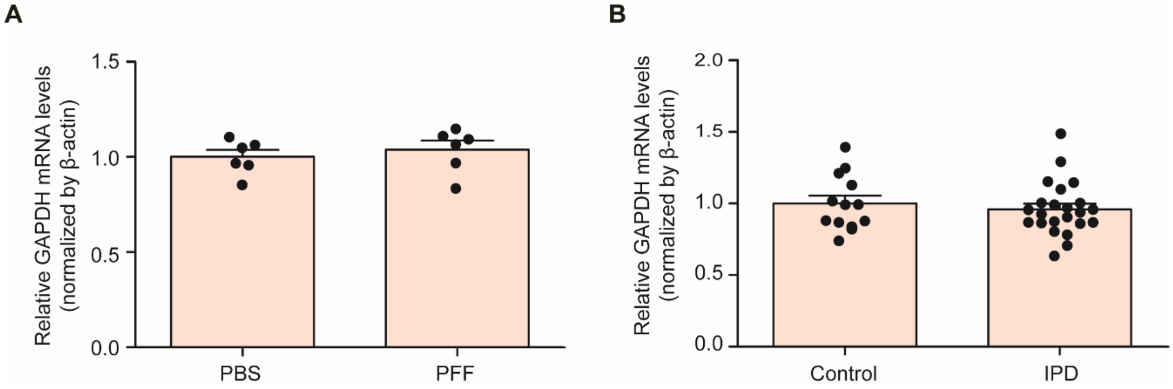

Figure S1. Relative GAPDH mRNA expression under PD relevant conditions. Relative expression levels of *GAPDH* gene transcript in RPMI-2650 cells treated with PBS (n = 6) or PFF (n = 6) (**A**), and in nasal fluid cells collected from control (n = 13) and PD patients (n = 23) (**B**) determined by reverse transcription quantitative PCR followed by normalization with β-actin.

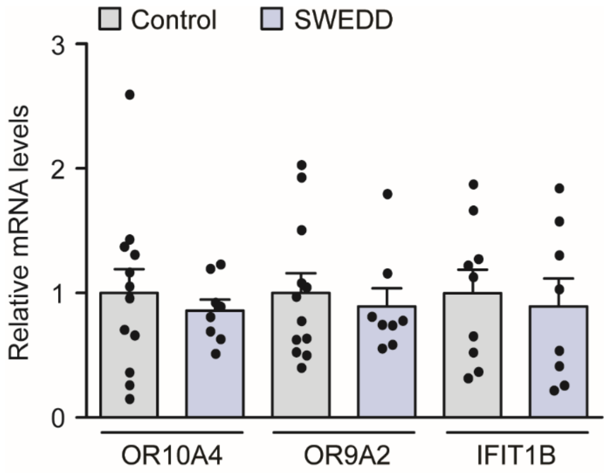

Figure S2. Levels of potential nasal PD biomarkers in SWEDD. Relative expression levels of *OR10A4*, *OR9A2*, *IFIT1B* gene transcripts in control vs. patients of SWEDD determined by reverse transcription quantitative PCR.

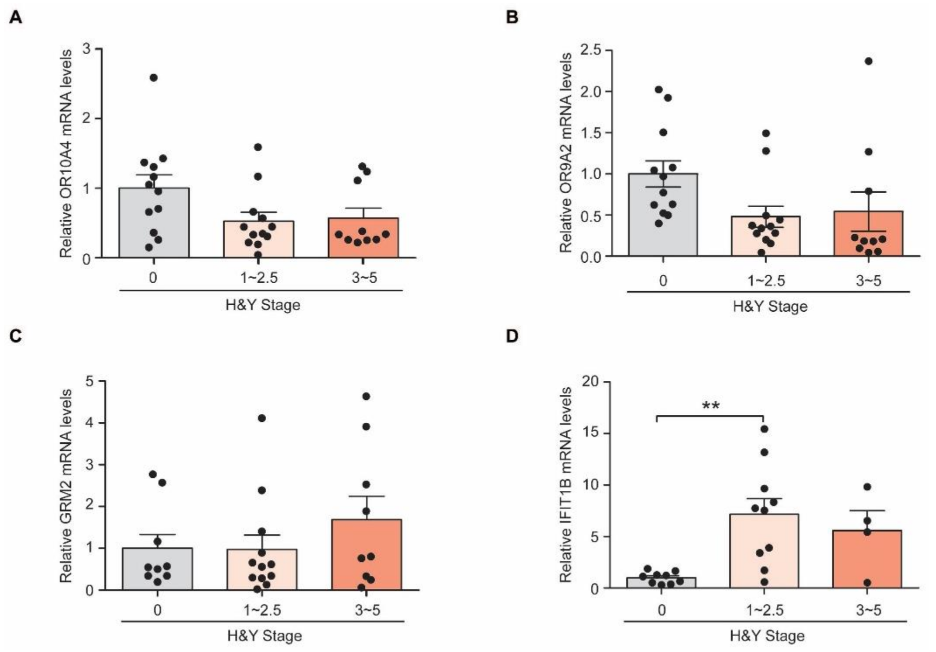

Figure S3. Levels of potential nasal biomarkers in different stages of PD. Relative expression levels of (**A**) *OR10A4*, (**B**) *OR9A2*, (**C**) *GRM2*, and (**D**) *IFIT1B* gene transcripts in control (stage 0) vs. patients of PD at different H&Y scale values (score ranges: 1–2.5 and 3–5) determined by reverse transcription quantitative PCR.

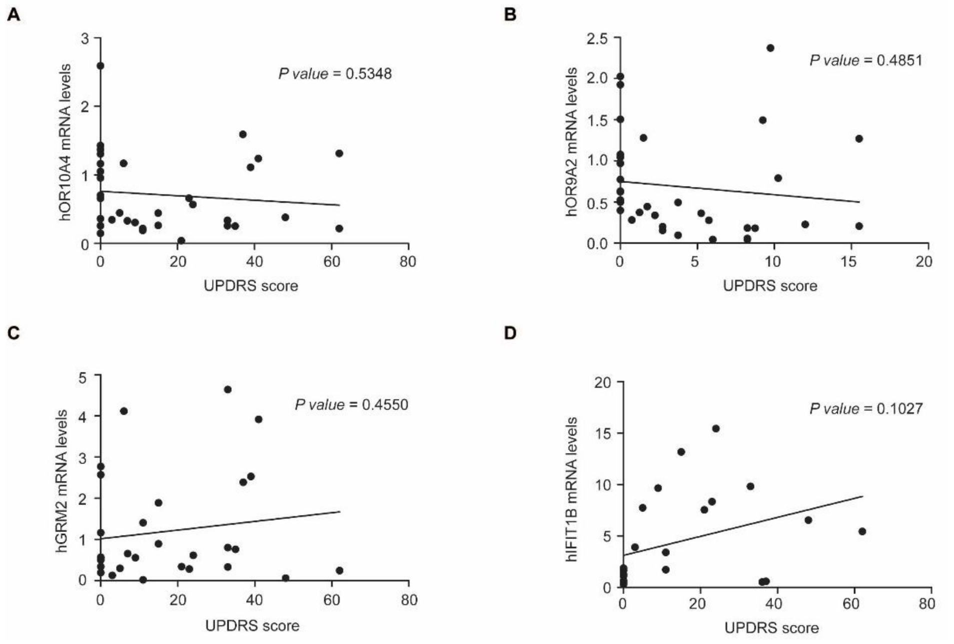

Figure S4. Correlation analyses of the levels of nasal *OR10A4*, *OR9A2*, *GRM2*, and *IFIT1B* and clinical UPDRS scores. (**A**–**D**) Correlation plots of the levels of nasal *OR10A4*, *OR9A2*, *GRM2*, and *IFIT1B* to UPDRS scores (n = 34 *OR10A4*, 34 *OR9A2*, 30 *GRM2*, and 23 *IFIT1B*). Pearson correlation analysis was performed between the level of each transcript and UPDRS scores.

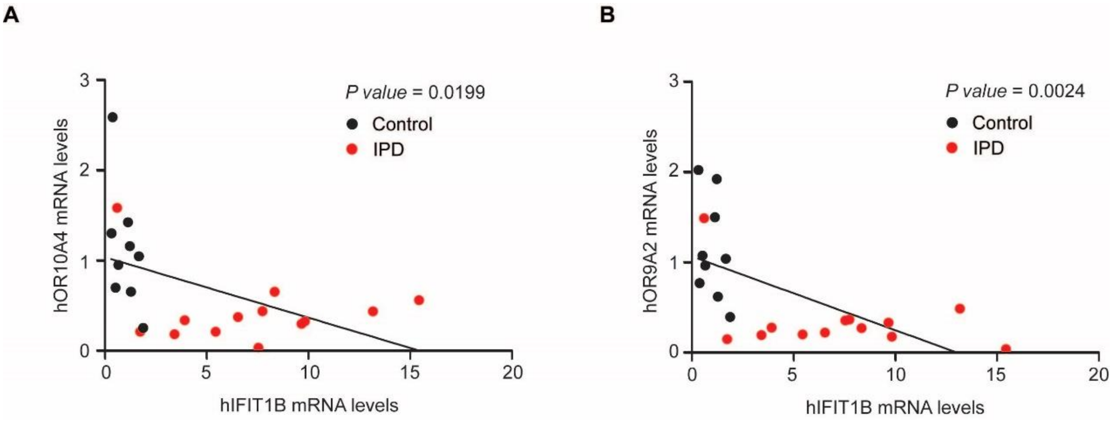

Figure S5. Correlation analysis between potential nasal biomarkers. (**A**) Pearson correlation analysis between the levels of nasal *OR10A4* and *IFIT1B* (n = 22 combined samples from the control and PD groups). (**B**) Pearson correlation analysis between the levels of nasal *OR9A2* and *IFIT1B* (n = 22 combined samples from the control and PD groups). Samples of the control and PD groups are colored in black and red, respectively, for easy comparison.

Table S1. Gene enrichment and functional annotation analysis using Cytoscape GlueGO (Gene ontology, biological process).

Please refer to the separate Exel file “Supplementary Table S1”.

Table S2. The complete list of genes with reproducible and more than 30% alteration between PFF-treated and PBS-treated RPMI-2650 cells.

Please refer to the separate Exel file “Supplementary Table S2”.
